# Relationship between three aspects of resilience—adaptive characteristics, withstanding stress, and bouncing back—in hospital workers exposed to prolonged occupational stress during the COVID-19 pandemic: a longitudinal study

**DOI:** 10.1186/s12913-023-09731-x

**Published:** 2023-06-28

**Authors:** Robert G. Maunder, Benjamin Rosen, Natalie D. Heeney, Lianne P. Jeffs, Jane Merkley, Kate Wilkinson, Jonathan J. Hunter, Jennie Johnstone, Rebecca A. Greenberg, Lesley A. Wiesenfeld

**Affiliations:** 1 Sinai Health, 600 University Ave., Toronto, M5G 1X5 Canada; 2 Department of Psychiatry, University of Toronto, Toronto, Canada; 3 Lunenfeld-Tanenbaum Research Institute, Sinai Health, Toronto, Canada; 4 Department of Laboratory Medicine and Pathobiology, University of Toronto, Toronto, Canada; 5 Department of Paediatrics, University of Toronto, Toronto, Canada

**Keywords:** Resilience, Healthcare workers, Nurses, Burnout, Longitudinal cohort study

## Abstract

**Background:**

The term resilience is used to refer to multiple related phenomena, including: (i) characteristics that promote adaptation to stressful circumstances, (ii) withstanding stress, and (iii) bouncing back quickly. There is little evidence to understand how these components of resilience are related to one another. Skills-based adaptive characteristics that can respond to training (as opposed to personality traits) have been proposed to include living authentically, finding work that aligns with purpose and values, maintaining perspective in the face of adversity, managing stress, interacting cooperatively, staying healthy, and building supportive networks. While these characteristics can be measured at a single time-point, observing responses to stress (withstanding and bouncing back) require multiple, longitudinal observations. This study’s aim is to determine the relationship between these three aspects of resilience in hospital workers during the prolonged, severe stress of the COVID-19 pandemic.

**Methods:**

We conducted a longitudinal survey of a cohort of 538 hospital workers at seven time-points between the fall of 2020 and the spring of 2022. The survey included a baseline measurement of skills-based adaptive characteristics and repeated measures of adverse outcomes (burnout, psychological distress, and posttraumatic symptoms). Mixed effects linear regression assessed the relationship between baseline adaptive characteristics and the subsequent course of adverse outcomes.

**Results:**

The results showed significant main effects of adaptive characteristics and of time on each adverse outcome (all *p* < .001). The size of the effect of adaptive characteristics on outcomes was clinically significant. There was no significant relationship between adaptive characteristics and the rate of change of adverse outcomes over time (i.e., no contribution of these characteristics to bouncing back).

**Conclusions:**

We conclude that training aimed at improving adaptive skills may help individuals to withstand prolonged, extreme occupational stress. However, the speed of recovery from the effects of stress depends on other factors, which may be organizational or environmental.

## Background

The COVID-19 pandemic has created extraordinary stress for healthcare workers [1, 2], a group who were experiencing high levels of burnout [3–6] and other mental health challenges [7–9] prior to the pandemic. Beyond the established impact of burnout on individuals [9–12] and on patient outcomes [12–14], this outcome of occupational stress may contribute to a widely reported shortages of nurses [15–18] and other healthcare professionals.

In the face of this severe occupational strain, resilience has received much attention. Indeed, as of this writing, a search reveals over 2,200 peer-reviewed papers that include both “COVID” and “resilience” in their titles. Individuals and organizations within the healthcare system seek to optimize resilience and minimize the harmful effects of occupational stress, including optimizing resilience in order to reduce the intention to quit healthcare work [19, 20], although it remains unclear how best to accomplish this [21].

This growing literature lacks focus, in part, because there is no accepted definition of the concept of resilience. Various definitions refer to (1) characteristics which are expected to promote successful adaptation in the face of adversity (hereafter referred to as “adaptive characteristics”) and/or (2) low levels of negative effects of adversity (“withstanding stress”), and (3) recovering rapidly and from negative effects (“bouncing back”).

Importantly, these are concepts that can be applied at various levels, from individuals to teams and large organizations, both within and beyond health care. Much of the empirical study of resilience in healthcare settings has focused on individuals, leading to large reviews [22–24], while studies of organizational resilience have been fewer [25].

Examples of definitions of resilience that emphasize adaptive characteristics include “the ability to positively adapt to traumatic or adverse experiences,” [26] the “ability to anticipate, prepare for and adapt to changing conditions,” [27] or, for organizations, the “capacity to adapt and respond to challenges and changes at different system levels” [28]. At an organizational level, characteristics that lead to resilience have been described as the optimal resolution of seven organizational drivers: (1) workload and job demands, (2) control and flexibility, (3) work-life integration, (4) meaning in work, (5) social support, (6) efficiency and resources, and (7) organizational culture and values [29]. In individuals, adaptive characteristics can be measured at a single time-point using self-report instruments. Such instruments measure a range of characteristics that have been considered to foster positive adaptation. The Connor-Davidson Resilience Scale has a five factor structure that includes (1) personal competence, high standards, and tenacity, (2) trust in one’s instincts, tolerance of negative affect, and endorsement of the strengthening effects of stress, (3) acceptance of change, and secure relationships, (4) sense of personal control, and (5) spiritual influences [30]. Some studies have focused on adaptive personality traits, which tend to be resistant to change [31]. Alternatively, the Resilience at Work scale, focusses explicitly on skills-based characteristics that are potentially teachable. It measures seven domains: (1) living authentically, (2) finding work that aligns with purpose and values, (3) maintaining perspective in the face of adversity, (4) managing stress, (5) interacting cooperatively, (6) staying healthy, and (7) building supportive networks [32]. Identifying mutable adaptive characteristics that are responsive to training is highly relevant to organizations that wish to enhance the resilience of their people and thus has practical advantages over measures of less mutable traits.

The second and third facets of the concept of resilience (withstanding stress and bouncing back) are dynamic, as they refer to the actual response of individuals or groups to adverse circumstances in a sequence of stress, response and recovery. Adaptive characteristics, which can be meaningfully measured at a single point in time prior to a stressful exposure, are distinct from the dynamic stress response, which must be observed over time. An example of a definition of resilience that includes these dynamic facets is “the ability to withstand, respond to and recover rapidly from disruption” [33]. Withstanding stress is often inferred from low scores on measures of adverse outcomes, such as burnout, psychological distress, or posttraumatic symptoms, following extraordinary stress. Quantifying success in bouncing back requires serial measures over time to identify the magnitude and rapidity of recovery, but longitudinal studies are much less common than single time-point cross-sectional studies. Longitudinal measures of stress response have been advocated for the study of pandemic-related stress and resilience for this reason [34].

For healthcare organizations, both quantitative and qualitative assessment of workers’ well-being has been suggested. Routine, repeated quantitative measurement of workers’ well-being has been suggested as an indicator of organizational resilience [35], while qualitative exploration at multiple organizational levels has identified the complexity of resilient performance [36].

**Fig. 1:**
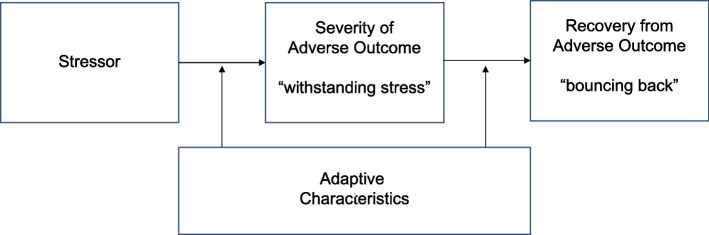
A conceptual model of the relationship between adaptive characteristics and resilient responses to a stressor

There has been little study of how these different aspects of resilience are related to one another. Theoretically, it would be expected that adaptive characteristics would buffer against disruption at the time of the stressor (contributing to withstanding stress) and lead to a more rapid and robust recovery from the stressor (contributing to bouncing back). A conceptual model incorporating these three aspects of resilience is provided in Fig. 1.

Longitudinal studies, most with a single follow-up time point, support the hypothesis of withstanding stress in some settings. For example, baseline adaptive characteristics are associated with lower reported stress and lower HbA1c levels at 1-year follow-up in diabetic patients [37] and with various longitudinal indicators of mental health in military personnel and veterans [38–41]. Similarly, during the COVID-19 pandemic, greater baseline adaptive characteristics have been associated with fewer mental health symptoms in the general population [42, 43] and lower self-reported stress in health care workers [44]. Personality characteristics, including higher agreeableness, conscientiousness, emotional stability and openness to new experiences, were associated with less severe depressive and anxiety symptoms three months later in health care workers [31].

Longer term follow-up, with measurement during and after stressful events, is required to test the hypothesis of bouncing back. To our knowledge the relationship between individual adaptive characteristics and bouncing back from a stressful exposure has not been studied in the context of a real-world occupational stress, particularly in healthcare workers. This is an important gap because bouncing back from the effects of stress rapidly and robustly is very desirable in a high stress environment and it would be useful to know whether to focus efforts to support individual resilience on training in skills-based adaptive characteristics or on some other target, such as organizational characteristics which have been identified as drivers of burnout or resilience.

The purpose of this study is to study the relationship between adaptive characteristics, withstanding stress, and bouncing back in healthcare workers. We use longitudinal data from seven time points in a single cohort of hospital healthcare workers working during COVID-19 in order to assess the relationship between adaptive characteristics measured early in the pandemic, and dynamic changes in adverse outcomes to occupational stress throughout the next 18 months. The adverse outcomes studied are those that are known to have been elevated in healthcare workers during the pandemic: burnout, psychological distress, and posttraumatic symptoms [45–47]. Adaptive characteristics in this study are selected to be those which are potentially modifiable through training. To assess the functional impact of adaptive characteristics, we also explore the relationship between adaptive characteristics at the start of the measurement period and thoughts of leaving one’s job 15 months later. This is particularly salient in time in which the healthcare force is depleted.

## Methods

### Study design and participants

This study took place in Toronto, Canada at an urban hospital that includes an acute care teaching hospital and a rehabilitation hospital with a combined staff of more than 6000. The survey methods for this study have been described previously [2]. Briefly, all staff (employees, physicians, learners, retail employees, volunteers, contractors) were invited via posters and emails to participate in a longitudinal study, completing a survey roughly every three months starting in fall 2020 until spring 2022. All participants provided informed consent. Surveys were completed using an online survey software (Alchemer, Louisville, CO) that is compliant with jurisdictional privacy standards, and received a gift card at the end of each completed survey (~ $20 CAD value). This study was performed in accordance with the Declaration of Helsinki and received approval from the Sinai Health Research Ethics board.

Eight hundred eighty-four participants consented to participate during the recruitment phase of the study, with 538 (61%) completing the first survey (T1, “fall 2020”), conducted from September 21-November 15, 2020, forming the cohort for further follow-up. Subsequent survey waves were conducted during: January 25-February 15, 2021 (T2, “winter 2021”); April 26-May 16, 2021 (T3, “spring 2021”); July 26-August 15, 2021 (T4, “summer 2021”); October 25-November 14, 2021 (T5, “fall 2021”); and January 24-February 13, 2022 (T6, “winter 2022”), April 25-May 16 2022 (“spring 2022”). The response rates for the subsequent surveys (the numerator calculated as the number of responses that included a valid measure of emotional exhaustion, psychological distress, or both) were: 485/538 (90%) at T2, 424/538 (79%) at T3, 409/538 (76%) at T4, 395/538 (73%) at T5, 372/538 (69%) at T6, and 350/538 (65%) at T7.

### Measures

Demographic data was self-reported by participants at T1. Occupational role was sorted into four categories based on a participant’s professional qualifications and their reported close patient contact (if they were within two metres of a patient for more than 15 min within the previous month). At T6, two questions were used to assess thoughts of leaving one’s work: “Are you considering leaving your job?” and (if yes) “Are you considering leaving healthcare?”.

Resilience was measured at T1 using the Resilience at Work Scale (RAW) [32], which was selected because it measures skills-based characteristics that are potentially trainable [32]. This instrument comprises 20 items which are separated into seven different subscales. Each item is rated on a Likert scale from 0–6 and standardized scores are calculated for each subscale in addition to a total RAW score. Standardized scores are expressed as a percentage of the maximum score, with higher scores reflecting higher levels of resilience[32]. The Cronbach’s ⍺ for this scale in our study was 0.87.

Burnout was measured at all time-points with the emotional exhaustion subscale of the Maslach Burnout Inventory-Human Services Survey for Medical Personnel (MBI) [48]. The MBI is measured on a Likert scale from 0–6 and contains three subscales: emotional exhaustion (9 items for a potential score of 0–54), depersonalization (5 items for a potential score of 0–30), and diminished personal accomplishment (8 items for a potential score of 0–48). In the current study, emotional exhaustion is analyzed as a continuous variable, but for reference, a commonly used cut-off indicating severe emotional exhaustion is a score of ≥ 27 [49]. Cronbach’s ⍺ over the course of the seven surveys ranged from 0.94 to 0.96.

The Impact of Events Scale – Revised (IES-R) is a 22-item questionnaire which assesses post-traumatic stress symptoms over the previous seven days in response to a specific traumatic event [50] – the COVID-19 pandemic in the case of this study. The IES-R is measured on a Likert scale of 0–4 and includes three subscales: intrusion, avoidance, and hyperarousal. A total score from a range of 0–88 is calculated. In the current study, the total IES-R score is analyzed as a continuous variable, but for reference, a score of ≥ 24 is considered meaningful and ≥ 33 is considered to indicate a probable PTSD diagnosis [50]. The IES-R was measured at time-points T1, T3, T5, and T7, with Cronbach’s ⍺ values of 0.95 to 0.96.

Psychological distress was also measured at all time-points using the Kessler K6, a 6-item scale with items scored from 0–4, yielding a range of 0–24 [51]. The K6 is able to discriminate between community cases and non-cases of psychiatric disorders with acceptable sensitivity and specificity [51]. In the current study, the total K6 score is analyzed as a continuous variable, but for reference, a cut-off of ≥ 13 is considered to indicate likely serious mental illness [52]. In this study, Cronbach’s ⍺ values for the K6 varied from 0.85 to 0.87.

### Analysis

Descriptive statistics were used to summarize participant characteristics. Continuous measures were summarized using means and standard deviations (*SD*), while categorical measures were summarized using counts and percentages. Bivariate analysis was completed using either Pearson’s correlation coefficient or a X^2^ test, as appropriate.

Variation in each adverse outcome over time was assessed in a mixed-effect linear regression model. Mixed effect models were used to test the effects of adaptive characteristics (the total Resilience at Work score entered as a continuous variable), time, and their interaction. In this analysis, withstanding stress is indicated by a main effect of adaptive characteristics on adverse outcomes, while bouncing back is indicated by an interaction between adaptive characteristics and time. An unstructured covariance structure was selected. Age and occupation type were included as covariates since lower age and less experience have been consistently identified as correlates of increased burnout [53–55]. and adverse psychological outcomes have been reported to be highest in nurses [21]. In order to illustrate differences in adverse outcomes over time at different levels of adaptive characteristics, data were plotted using adaptive characteristics categorized into three equal terciles (lower, medium, higher) using cut-offs of RAW scores of 63.3 and 74.2. These tercile were also used to test the association between adaptive characteristics and the intention to leave work by X^2^ test. All analyses were performed with IBM SPSS Statistics 28 (Arnock, New York) and R (v 4.2.1) [56].

Statistical power was estimated for a planned analysis using repeated measures ANOVA. Since the analytic method finally chosen (mixed-effect linear regression) was expected to have greater statistical power than ANOVA, the power analysis is conservative. We calculated the effect size detectable in repeated measures ANOVA with the available sample (assuming *n* = 280 participants with data at all points to be included). For repeated measures ANOVA involving three groups and measurement at seven time points, setting significance at 0.05, and with a correction for non-sphericity consistent with that found in our sample (ε = 0.8), a sample of 280 people has 80% power to detect weak effects (as small as *f* = 0.24 for within-group differences, *f* = 0.21 for between-group differences, and *f* = 0.27 for interactions). Power calculation was performed with WebPower (www.webpower.psychstat.org/models/means05/ [56]).

## Results

The characteristics of participants at each time point are summarized in Table 1.

**Table 1:** Characteristics of the sample

Demographic	T1 *n* = 538	T2 *n* = 485	T3 *n* = 424	T4 *n* = 409	T5 *n* = 395	T6 *n* = 372	T7 *n* = 350
*Occupation Type*^a^							
Nurses & nursing students	134	118	98	100	95	89	84
Other clinical professionals	156	136	129	114	117	112	102
Other clinical personnel	90	85	69	69	65	59	59
Non-clinical personnel	158	146	128	126	118	112	105
*Gender (missing 1 – all time-points)*							
Female	422	385	358	341	326	311	291
Male	85	71	60	57	62	54	51
Other/Prefer not to say	30	28	5	10	6	6	7
*Age*							
Mean (*SD*)	38.5 (11.9)	38.5 (12.0)	39.2 (11.6)	38.8 (11.6)	39.1 (11.5)	39.2 (11.2)	39.1 (11.4)
Range	18–76	18–76	18–76	18–76	18–76	18–75	18–75
*Ethnic Group (missing 1 – T1, T2, T4)*							
African/Black	30	25	19	19	14	15	14
Asian	148	132	118	120	117	113	105
East Indian	35	32	28	27	28	24	25
European/White	278	254	223	209	202	189	174
Hispanic	15	12	7	6	6	6	5
Other/Mixed/Multiple	31	29	29	27	28	25	27

Other clinical professionals: Physician, resident, dietician, occupational therapist, social worker, physiotherapist, manager of clinical area, speech language pathologist, pharmacist, respiratory therapist, spiritual care practitioner

Other clinical positions: Administrative assistant, medical imaging technologist, manager, retail employee, clerk, porter, research staff, assistant to physician/occupational therapist/physiotherapist, housekeeper, administrator, volunteer

Non-clinical positions: Research scientist, research staff, laboratory technician, corporate and administrative staff, administrative assistant, volunteer, manager of non-clinical area, building services staff, clerk, laboratory technologist, housekeeper, retail employee

^a^Specific job types, in descending order of number of participants based on results at T1. Groups with two or fewer members not listed. Some roles appear in both clinical and non-clinical lists as determined by patient contact as described by participant

Among 522 survey participants who completed the Resilience at Work measure of adaptive characteristics at T1, the mean score was 68.1 (*SD* 11.9). There was no difference in adaptive characteristics by gender, ethnic group or occupation type (data not shown). Older age was significantly but weakly associated with better adaptive characteristics (*R* = 0.15, *p* < 0.001). Adaptive characteristics did not differ between participants who completed T7 measures (*n* = 341, *M* 67.8, *SD* 12.2) and those who had stopped participating before this (*n* = 181, *M* 68.5, *SD* 11.3, *F* (*df* 1) = 0.5, *p* = 0.48).

Regarding trends in adverse outcomes over time, for each of emotional exhaustion, psychological distress, and posttraumatic symptoms, there was a significant main effect of adaptive characteristics, indicating that this measure was related to adverse outcomes throughout the study. There was also a significant main effect of time on each adverse outcome. However, there was no significant interaction of adaptive characteristics and time for any of the adverse outcomes, indicating that different levels of adaptive characteristics did not predict differences in the rate of change of outcomes over time, as would be expected if adaptive characteristics were associated with “bouncing back” (Table 2).

**Table 2:** The relationship between adaptive characteristics, time, and their interaction on adverse outcomes to stress over 18 months of working during a pandemic

**Fixed Effects**	**Emotional exhaustion**			**Psychological distress**			**Posttraumatic symptoms**		
	Estimates	CI	*p*	Estimates	CI	*p*	Estimates	CI	*p*
(Intercept)	63.44	57.22 – 69.65	< 0.001	21.96	19.81 – 24.11	< 0.001	61.26	52.79 – 69.73	< 0.001
Resilience (RAW)	-0.49	-0.57 – -0.41	< 0.001	-0.17	-0.20 – -0.14	< 0.001	-0.42	-0.54 – -0.31	< 0.001
Time [T2]	0.95	-4.15 – 6.05	0.72	-1.59	-3.54 – 0.36	0.11			
Time [T3]	0.59	-4.62 – 5.80	0.82	0.12	-1.87 – 2.10	0.91	-3.71	-11.84 – 4.43	0.37
Time [T4]	-0.73	-5.96 – 4.49	0.78	-2.49	-4.48 – -0.49	0.01			
Time [T5]	-1.41	-6.64 – 3.82	0.60	-2.05	-4.04 – -0.06	0.04	-13.05	-21.23 – -4.86	0.002
Time [T6]	-2.26	-7.66 – 3.14	0.41	-1.76	-3.81 – 0.29	0.09			
Time [T7]	-1.85	-7.30 – 3.60	0.51	-1.81	-3.89 – 0.27	0.09	-9.74	-18.29 – -1.19	0.03
RAW^*^Time [T2]	0.03	-0.05 – 0.10	0.50	0.03	0.00 – 0.06	0.03			
RAW^*^ Time [T3]	0.03	-0.04 – 0.11	0.40	0.02	-0.01 – 0.04	0.31	0.07	-0.05 – 0.19	0.25
RAW^*^ Time [T4]	0	-0.07 – 0.08	0.91	0.03	0.00 – 0.06	0.03			
RAW^*^ Time [T5]	0.02	-0.05 – 0.10	0.56	0.03	0.00 – 0.06	0.05	0.16	0.04 – 0.27	0.01
RAW^*^ Time [T6]	0.06	-0.02 – 0.14	0.13	0.04	0.01 – 0.07	0.02			
RAW^*^ Time [T7]	0.04	-0.04 – 0.12	0.37	0.03	0.00 – 0.06	0.03	0.1	-0.02 – 0.23	0.11
**Random Effects**									
σ^2^	45.87			6.64			110.25		
τ_00_ _Participant.ID_	81.59			8.79			125.2		
ICC	0.64			0.57			0.53		
N _Participant.ID_	517			517			515		
Observations	2854			2834			1628		
Marginal R^2^ / Conditional R^2^	0.242 / 0.727			0.243 / 0.674			0.144 / 0.599		

**Fig. 2:**
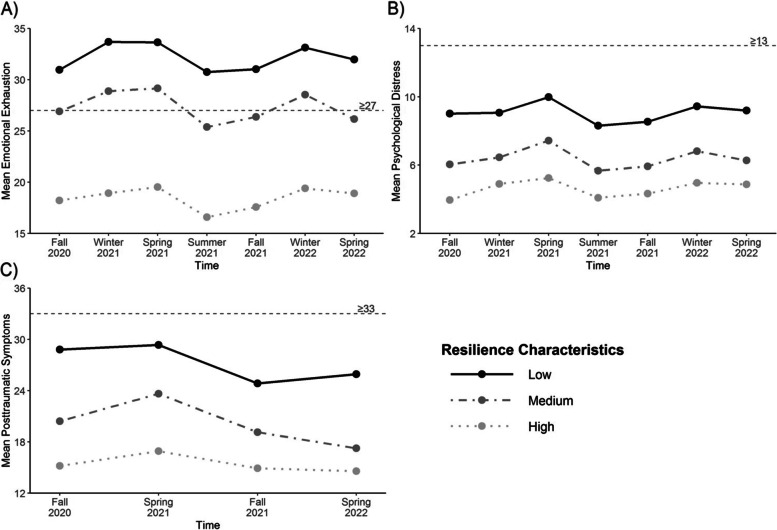
Temporal patterns of outcome variables in groups with low, medium or high resilience characteristics

For each outcome, the direction of the association was that greater adaptive characteristics were associated with lower severity of adverse outcomes. This is illustrated in Fig. 2. Greater adaptive characteristics measured at T1 are associated with significantly lower emotional exhaustion (Panel A), lower psychological distress (Panel B), and lower posttraumatic symptoms (Panel C) at each time point.

The clinical significance of this effect is illustrated by the difference in mean values of adverse outcomes between individuals in the lowest tercile of adaptive characteristics and those in the highest tercile. For emotional exhaustion, the former group has mean values > 30 at each time point (higher than the cut-off of 27 that has been used to define severe emotional exhaustion [57, 58]). In contrast, mean values of emotional exhaustion for individuals in the highest tercile of adaptive characteristics were < 20 at each time point (under the lower cut-off of 21 that has been used to define high emotional exhaustion). The lowest tercile of adaptive characteristics had mean values of psychological distress ranging from 8.1 to 10.0 (which can be compared to the screening cut-off of 13 for “likely serious mental disorder” [59]), with means in the highest tercile of adaptive characteristics about 25% lower (6.1 to 7.4). The lowest tercile of adaptive characteristics had mean values of posttraumatic symptoms in the range of 23.6 to 28.9 (which can be compared to the screening cut-off of 33 for likely cases of PTSD [60]), whereas highest tercile of adaptive characteristics had values about 20% lower (19.2 to 22.6).

However, adaptive characteristics were not associated with temporal changes in any of these outcomes from time point to time point (i.e. the slope of the line).

Regarding a potential functional impact of adaptive characteristics, Table 3 indicates a significant association of adaptive characteristics at the start of the study and consideration of leaving one’s job 15 months later (X^2^ (*df* 4) = 25.8, *p* < 0.001). Of those in the highest tercile of adaptive characteristics, 20% were considering leaving their current job or leaving healthcare, whereas in the lowest tercile 48% reported considering these options.

**Table 3:** Association between adaptive characteristics at T1 and consideration of leaving job at T6

Thoughts about leaving work		Adaptive characteristics		
		Lowest tercile	Middle	Highest tercile
	*n*	119	125	115
Not considering leaving	246	62, 52%	91, 73%	93, 81%
Considering leaving current job, not healthcare	52	23, 19%	18, 14%	11, 10%
Considering leaving healthcare	61	34, 29%	16, 13%	11, 10%

## Discussion

This longitudinal cohort study demonstrates that among healthcare workers performing a broad range of roles at two hospital sites over 15 months of the COVID-19 pandemic, adaptive characteristics measured early in the pandemic were associated with withstanding subsequent stress, with respect to outcomes of psychological distress, posttraumatic symptoms, and emotional exhaustion. Differences in the severity of these adverse outcomes between those higher and lower in adaptive characteristics are large enough to be clinically significant. On the other hand, adaptive characteristics were not associated with bouncing back from stress effects more completely or more rapidly.

The lack of association between adaptive characteristics of individuals and temporal patterns of stress outcomes indicates that different aspects of resilience may be relatively independent of one another and emphasizes the importance of identifying meaningful metrics of each aspect of resilience. The dynamic rise and fall of adverse outcomes for healthcare workers experiencing a prolonged and fluctuating stressor is only apparent when measurement occurs at repeated intervals. Indeed, routine surveillance of healthcare workers’ well-being has been advocated as a leading indicator of organizational resilience [35], which could be used for early identification of problematic trends and targeted intervention for local “hot-spots.”

Conceptually, differentiating withstanding stress from bouncing back is a trajectory-based description of stress responses and thus is related to the trajectories of response to major life events that have been described previously by Bonanno [61]. Among these response trajectories, withstanding stress corresponds to the trajectory labelled resilience, while bouncing back corresponds to the trajectory labelled recovery. Recovery is noted to be the modal response to a singular traumatic event [62]. The current study does not concern a discrete event but rather a dynamically changing and prolonged stressor, which complicates the assessment of trajectories. The overall pattern of responses over time in our study is consistent with the capacity of healthcare workers to recover from perturbations in adverse psychological outcomes, since the severity of these outcomes rise and fall, presumably in response to the rising and falling severity of stressful events. We do not find evidence, however, that this capacity for recovery, or bouncing back, is related to individual adaptive characteristics.

Note that the analysis had sufficient sample size to detect weak interactive effects. Statistically significant associations between the resilience*time interaction and psychological outcomes (psychological distress and posttraumatic symptoms but not emotional exhaustion) were weak and inconsistently present at different time points, which does not support an overall contribution of resilience characteristics to recovery.

Although individual adaptive characteristics were not associated with bouncing back, they were strongly related to withstanding occupational stress. This is a hopeful finding, given that the adaptive characteristics measured by the Resilience at Work instrument were explicitly chosen by its developers to be modifiable skills or strategies that can be taught [63]. Given their strong relationship with withstanding stress, teaching these skills may be a valuable organizational strategy for supporting the well-being of healthcare workers facing extraordinary stress. In this regard, it is noteworthy that the inter-correlations of the dimensions measured in the Resilience at Work scale were not high (ranging from 0.12 to 0.55 with a median inter-correlation of 0.27, full data not shown), which is consistent with a scale largely composed of distinct skills, which might require distinct types of training. It also raises the possibility for future research of determining if specific aspects of adaptive function differ in their relationships to burnout trajectories.

The association of low adaptive characteristics with thoughts of leaving one’s job late in the pandemic emphasizes the functional relevance of occupational stress and resilience for the healthcare system. Although considering leaving one’s job need not necessarily translate into action, the consistently reported trend that healthcare workers, especially nurses, were indeed choosing to leave their jobs or their profession during the pandemic [16, 17, 64, 65], suggests that this consideration should be taken seriously.

Conceptually, the results of this study argue for distinguishing between different facets of individual resilience. The magnitude of the severity of adverse outcomes (withstanding stress) was distinct from the temporal pattern of recovery (bouncing back) when adverse outcomes were measured serially over time. Not only can these aspects of resilience be measured separately, they appear to respond to different drivers. Thus, the results of this study support multi-faceted definitions of individual resilience, which combine (at least) three separate constructs: characteristics that are relevant to adaptation, a response to stress, and a trajectory of recovery.

Although longitudinal studies of adverse psychological outcomes in healthcare workers during the pandemic have been reported [66–68], few have assessed the relationship of these outcomes to adaptive characteristics. One, reporting on a 3-month period early in the pandemic, found that an investigator-authored 6-item measure of adaptive characteristics (perceptions of workplace stress, support, positive affect experienced prior to COVID-19, frequency of working nontraditional shifts, and frequency of sleep disturbances) was unrelated to IES-R scores one and three months later [69]. The difference between this result and the current study could be due to the nature of the measure of adaptive characteristics, the different populations measured, or differences in the impact of working early and later in the pandemic on posttraumatic symptoms.

It is noteworthy that other studies identify organizational factors associated with resilience that were not assessed in this study, including optimizing workload and job demands, control and flexibility, work-life integration, the meaningfulness of work, social support, organizational culture and values, and workplace safety, as well as providing focused training in novel tasks (for example during redeployment), and maintaining visible, responsive, authentic leadership [29, 70–72]. While a large literature has developed regarding interventions that are recommended in preparation for and during a major occupational stress [73], less is known about the distinct needs of healthcare workers during the recovery phase, particularly given the unprecedented magnitude and duration of stress related to the COVID-19 pandemic. Ongoing surveillance of staff well-being may be a useful indicator of which interventions prove to be more and less successful.

The implications of this study are that skills-based adaptive characteristics, which are potentially mutable through training, are strongly related to one aspect of resilience, withstanding stress, in the context of prolonged, severe occupational stresses which fluctuate in intensity. This suggests that providing such training may be a valuable preventive intervention that is available to healthcare organizations. Testing the efficacy of such interventions in appropriately controlled prospective research studies is warranted. On the other hand, this study provides little evidence that adaptive characteristics are related to bouncing back from the adverse effects of stress more quickly or more completely. The only exception to this null finding in the current study is a near-significant trend that suggests the possibility that larger studies could find a weak relationship between adaptive characteristics and bouncing back from posttraumatic symptoms. Thus, if bouncing back more quickly is possible in circumstances such as those studied here, our results suggests that future research must look to other variables (such as workplace characteristics) for potential mediators.

The strengths of this study include its repeated measurement of relevant variables using validated instruments over 18 months of an extraordinary historical stressor, its inclusion of participants with a diverse variety of occupational roles within healthcare, and its relatively large sample size. Limitations include the method of recruitment, which did not include a sampling strategy ensuring a representative sample. The retention rate over six measurement points, while a strength compared to many study of healthcare workers in which response rates and retention are much lower, nonetheless is susceptible to the possibility that those who chose to stop participating differed from those who continued in important ways (such as experiencing greater burnout, or finding questions about well-being and stress to lack personal salience). It is noteworthy in this respect that adaptive characteristics measured at baseline did not differ between participants who completed T7 measures and those who dropped out earlier. Furthermore, the dynamic variability of the severity of occupational stressors during the study period was very likely to be substantial but was unmeasured. Experientially, the severity of stress changed in relation to fluctuations in workload and personal risk as case numbers rose and fell in waves and public health measures changed.

## Conclusions

In summary, the results of this longitudinal cohort study suggest that individual adaptive characteristics have been strong buffers of the severity of adverse responses to occupational stress among healthcare workers in COVID-19. The rate of recovery from severe occupational stress, on the other hand, appears to depend on other factors. Determining how to optimize the speed and magnitude of recovery of healthcare workers after the COVID-19 pandemic remains an urgent goal of healthcare systems.

## Data Availability

The dataset analysed during the current study is not publicly available because the participants have not provided consent for the publication of individual data but a de-identified dataset is available from the corresponding author on reasonable request.
